# CT-Like MRI for Assessing Shoulder Bone and Ligament Injuries

**DOI:** 10.7759/cureus.84068

**Published:** 2025-05-13

**Authors:** Dai Tajima, Shinji Yamamoto, Masashi Imao, Hiromi Watanabe

**Affiliations:** 1 Department of Radiological Technology, Japan Community Health Care Organization (JCHO) Saitama Medical Center, Saitama, JPN; 2 Department of Radiological Technology, Japan Community Health Care Organization (JCHO) Tokyo Yamate Medical Center, Tokyo, JPN; 3 Department of Radiological Technology, Gunma Paz University, Takasaki, JPN; 4 Department of Radiology, Japan Community Health Care Organization (JCHO) Saitama Medical Center, Saitama, JPN

**Keywords:** bone morphology, ct-like imaging, ligament evaluation, mri, shoulder joint

## Abstract

Introduction

This study investigates the use of “CT-like imaging” derived from a 3D multi-echo gradient-recalled echo (GRE) MRI sequence to assess the morphology of bones and ligaments in the shoulder joint.

Methods

CT-like images were produced using a 3D multi-echo GRE sequence with optimized imaging parameters, including a 5° flip angle and the combination of two echo acquisitions. A monopolar readout gradient was employed to minimize ligament blurring, and subtraction processing was applied to enhance the visualization of both bone and ligament structures.

Results

In a clinical case of anterior shoulder dislocation, the CT-like images successfully revealed bone injuries with clarity comparable to that of conventional CT. In addition, this MRI-based technique provided superior visualization of ligament structures.

Conclusions

This radiation-free imaging approach offers particular advantages for radiosensitive patients. However, further validation in multicenter studies across various age groups is necessary to establish its broader clinical utility.

## Introduction

The shoulder joint has the greatest range of motion in the human body and is also the most commonly dislocated joint, accounting for approximately 45% of all dislocations [1]. Among these, anterior shoulder dislocations constitute the vast majority, with an incidence of around 95% [2]. Such dislocations are often associated with complications, including Hill-Sachs and Bankart lesions, fractures of the greater or lesser tuberosity, humeral neck fractures, and injuries to the rotator cuff or surrounding nerves [3-7].

Both arthrographic CT and MRI have demonstrated clinical value in evaluating ligamentous and labral injuries [8,9]. While MRI can detect bone lesions, CT remains the gold standard for assessing bone morphology due to its superior spatial resolution and clarity [10,11]. However, the use of ionizing radiation limits its suitability, particularly for radiosensitive populations.

Recent advancements in MRI hardware and imaging protocols have led to the emergence of “CT-like” MRI techniques. These approaches aim to replicate the image quality of CT for bone assessment while avoiding radiation exposure. One such method is Fast Field Echo Resembling a CT Using Restricted Echo-spacing (FRACTURE), developed by Johnson et al. [12], which employs a multi-echo gradient-recalled echo (GRE) sequence combined with grayscale inversion to enhance bone contrast. Similar techniques have been introduced under different manufacturer-specific names.

Despite their promise, CT-like MRI methods have been underutilized in the evaluation of the shoulder joint - a complex anatomical region with overlapping musculature, bones, and ligaments. Importantly, prior studies have not established the optimal MRI conditions (e.g., flip angle, echo combination, and gradient polarity) that best enhance contrast and minimize artifacts for this region. Imaging parameters are especially critical for visualizing ligaments, which typically have short T2* values and appear hypointense on conventional MRI.

In this preliminary study, we aimed to identify the optimal MRI acquisition parameters for CT-like imaging to improve the visualization of shoulder bones and ligaments. Our primary objective was to evaluate image quality and signal characteristics under different imaging settings. As a secondary goal, we assessed the clinical feasibility of this radiation-free technique in a representative case of anterior shoulder dislocation. Given the limited sample size and single-case clinical application, this study is exploratory and intended to lay the groundwork for future investigations involving larger and more diverse populations.

## Materials and methods

Study design

This prospective, single-center study was conducted to evaluate the feasibility and optimize the imaging parameters of CT-like MRI for assessing the shoulder joint. The study was carried out in two phases. In the first phase, MRI scans were performed on healthy volunteers to systematically identify optimal imaging settings using both quantitative metrics - such as signal-to-noise ratio (SNR), contrast-to-noise ratio (CNR), and full width at tenth maximum (FWTM) - and qualitative image assessments. In the second phase, the optimized protocol was applied to a clinical case to assess its effectiveness in visualizing bone and ligament structures.

Ethical approval

The study was approved by the Ethics Committee of the Japan Community Health Care Organization Saitama Medical Center (approval number 24-14). Written informed consent was obtained from all participants, which included 11 healthy volunteers and one clinical patient. To avoid unnecessary radiation exposure, no CT scans were performed on the volunteer group. For the same ethical reasons, enrollment was limited to participants aged 50 years or younger.

Participants

The study population consisted of 11 healthy volunteers (mean age: 32 ± 8 years; eight men and three women) and one clinical case - a 40-year-old woman with an anterior shoulder dislocation. Given the exploratory nature of the study, the sample size was intentionally limited to assess feasibility and optimize imaging parameters rather than establish clinical generalizability.

Imaging protocol

All MRI examinations were conducted using a 1.5T scanner (MAGNETOM Altea, Siemens, Erlangen, Germany) equipped with an 18-channel matrix body array coil. A 3D multi-echo GRE sequence was used. CT-like MRI images were generated through grayscale inversion and subtraction processing. Detailed imaging parameters are provided in Table 1.

**Table 1:** Sequence parameters for MRI at 1.5T GRE, gradient-recalled echo; TE, echo time; TR, repetition time

Parameter	Value
Protocol	3D multi-echo GRE
Orientation	Axial
TR (ms)	20
TE (ms)	4.76, 9.53, 14.29, 19.06
Slice thickness (mm)	1
Field of view read (mm)	200
Matrix (phase × read)	224 × 224
Slices per slab	160
Distance factor (%)	20
Averages	1
Fat suppression	None
Parallel imaging factor	2 (in phase-encoding direction)
Phase oversampling (%)	50
Slice oversampling (%)	14.3
Bandwidth (Hz/pixel)	380

Objective image quality assessment

**Figure 1:**
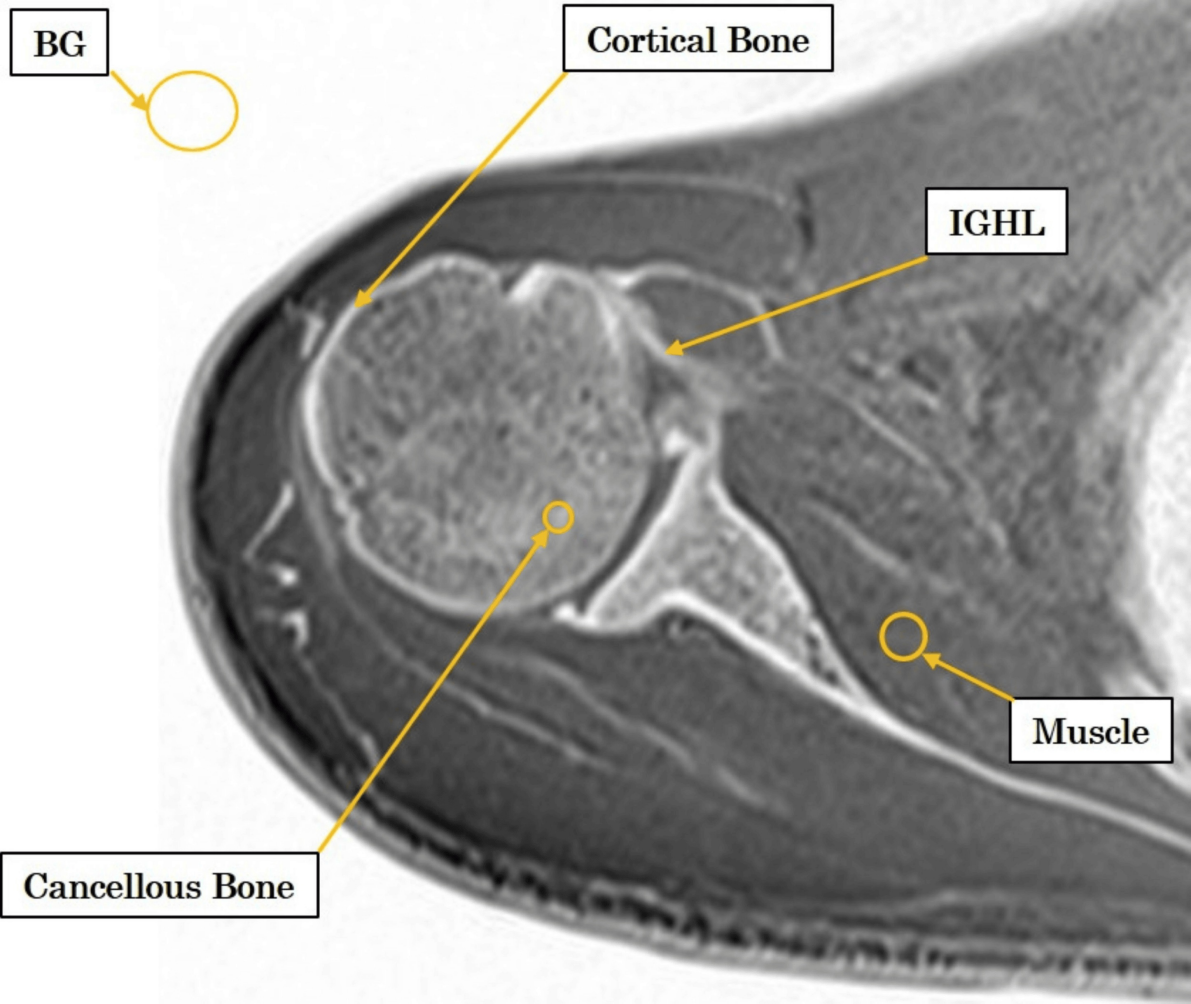
ROI positions used for image analysis ROIs were placed on the IGHL, subscapularis muscle, trabecular bone, and cortical bone to calculate the SNR, CNR, and FWTM. BG, background; CNR, contrast-to-noise ratio; FWTM, full width at tenth maximum; IGHL, inferior glenohumeral ligament; ROI, region of interest; SNR, signal-to-noise ratio

To identify the optimal conditions for CT-like imaging, the SNR was measured for the inferior glenohumeral ligament (IGHL), cortical bone, trabecular bone, and subscapularis muscle (Figure 1).

To determine the optimal conditions for CT-like imaging, SNR was measured in the IGHL, cortical bone, trabecular bone, and subscapularis muscle (Figure 1). Mean signal intensities and mean background signal were used to calculate SNR and CNR. The SNR was calculated using the formula √(π/2) × Ms / Mb, and the CNR was calculated as √(π/2) × (Ms₁ − Ms₂) / Mb, where Ms₁ and Ms₂ represent the mean signal intensities of the structure and the reference tissue, respectively.

To assess image sharpness, the FWTM was calculated from IGHL profile curves generated under both monopolar and bipolar gradient conditions. Gradient polarity was adjusted to evaluate the impact of chemical shift artifacts.

**Figure 2:**
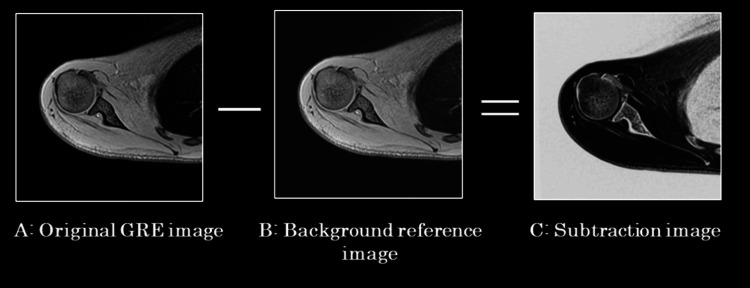
Diagram of subtraction image processing Subtraction images were generated using the formula C = A − 2B, where A is the original GRE image containing both target and background signals, and B is a background reference image, typically acquired from a later echo or from a region with high fluid or muscle signal. The resulting subtraction image (C) improves contrast by eliminating non-target background signals and enhances the visibility of low-intensity structures such as cortical bone and ligaments.

Subtraction images were generated using the equation C = A − 2B, where A represents the original GRE image and B is a background reference image, typically obtained from a later echo or from a region with high signal, such as fluid or muscle. The resulting image C was used to suppress non-target signals and enhance contrast while avoiding negative intensity values, thereby improving the visualization of low-signal structures such as cortical bone and ligaments (Figure 2).

Flip angle and echo combination optimization

Flip angles of 5°, 10°, 15°, and 20° were evaluated using in-phase echo times (TEs) ranging from 4.76 to 19.06 ms. Combined echo images, incorporating one to four echoes, were generated and analyzed for SNR and CNR. A flip angle of 5° combined with two echoes was determined to be optimal, offering the highest CNR and favorable visual assessment scores without inducing signal inversion between trabecular bone and muscle.

Readout gradient polarity optimization

To evaluate the impact of gradient polarity on ligament visualization, monopolar and bipolar readout gradients were compared. The FWTM of the IGHL profile was used as an objective metric, with lower values indicating reduced signal dispersion and sharper ligament depiction.

Evaluation of subtraction imaging

The effectiveness of subtraction processing was assessed by comparing the CNR between the IGHL and subscapularis muscle, as well as FWTM values, in original and subtracted images. Subtraction processing enhanced contrast while maintaining clear ligament edge definition.

Visual assessment

Axial images obtained under each imaging condition were independently reviewed by 15 medical professionals, including orthopedic surgeons, radiologists, and radiologic technologists with three to 20 years of experience. Image quality was scored using a five-point scale (Table 2), with higher scores reflecting better overall image quality.

**Table 2:** Visual assessment criteria IGHL, inferior glenohumeral ligament

Score	Criterion
5	IGHL, cortical bone, and trabecular bone can be evaluated with a high degree of confidence.
4	IGHL and cortical and trabecular bone are well delineated, allowing for reliable image evaluation.
3	IGHL and cortical bone are clearly visible, but trabecular bone visualization is slightly limited. Coarse pathological changes can still be assessed.
2	IGHL and cortical bone are moderately unclear, and trabecular bone is poorly visualized. Diagnostic confidence is low.
1	Image quality is insufficient for evaluation.

Clinical application

Using the optimized protocol (5° flip angle, two combined echoes, monopolar readout, and subtraction processing), a clinical case of anterior shoulder dislocation was scanned. The MRI acquisition parameters were as follows: repetition time (TR) = 20 ms, echo times (TE) = 4.76, 9.53, 14.29, and 19.06 ms; slice thickness = 1 mm; field of view = 200 mm; matrix size = 224 × 224; slices per slab = 160; distance factor = 20%; averages = 1; bandwidth = 380 Hz/pixel; parallel imaging factor = 2 (phase encoding direction); phase oversampling = 50%; and slice oversampling = 14.3%.

CT-like MRI findings were compared with conventional CT for evaluating bone injuries in the humeral head and glenoid, and with T2-weighted MRI for assessing the IGHL and surrounding soft tissues.

Statistical analysis

All statistical analyses were performed using EZR software (version 1.60) [13]. The Wilcoxon rank-sum test with Bonferroni correction was used for multiple comparisons. A p-value of <0.05 was considered statistically significant.

## Results

Optimal imaging conditions: flip angle and number of combined echoes

**Figure 3:**
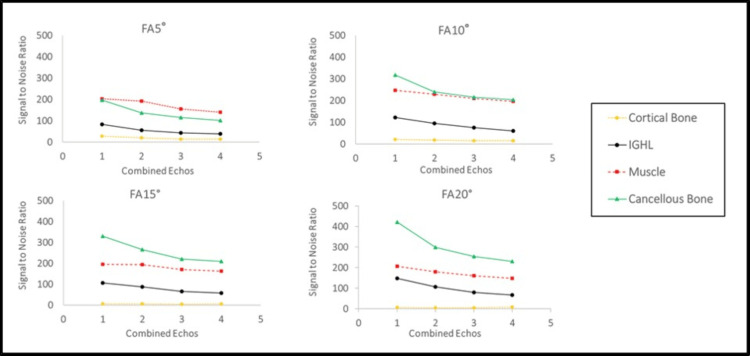
SNR changes across different flip angles and combined echoes Signal inversion between trabecular bone and subscapularis muscle was observed at flip angles ≥10°. FA, flip angle; IGHL, inferior glenohumeral ligament; SNR, signal-to-noise ratio

Figure 3 shows the SNRs for the IGHL, trabecular bone, and subscapularis muscle across varying flip angles (5°, 10°, 15°, and 20°) and different numbers of combined echoes (1 to 4).

In general, SNRs decreased with an increasing number of echo combinations for all tissues. At a 5° flip angle, the SNR of trabecular bone remained lower than that of the subscapularis muscle across all echo combinations (trabecular bone: 196.5, 137.2, 114.8, 101.5; subscapularis muscle: 202.8, 192.3, 155.3, 140.3 for echoes 1-4, respectively), indicating no signal inversion.

In contrast, at flip angles ≥10°, the SNR of trabecular bone exceeded that of the subscapularis muscle. Specifically, at a 10° flip angle, the SNR values were as follows: trabecular bone = 317.1, 240.1, 214.7, 202.9; subscapularis muscle = 246.7, 228.9, 209.8, 196.4. At 15°: trabecular bone = 265.8, 201.5, 185.4, 171.2; subscapularis muscle = 215.1, 192.2, 170.6, 158.3. At 20°: trabecular bone = 238.7, 188.3, 171.0, 160.4; subscapularis muscle = 192.5, 173.1, 158.9, 149.7. This inversion trend highlights an atypical contrast behavior under GRE imaging, and thus, flip angles ≥10° were excluded from further CNR and visual analyses.

The highest SNR for trabecular bone was observed at a 10° flip angle with a single echo (317.1 ± 20.2), while the highest SNR for the subscapularis muscle was observed at 5° with a single echo (202.8 ± 15.4).

**Figure 4:**
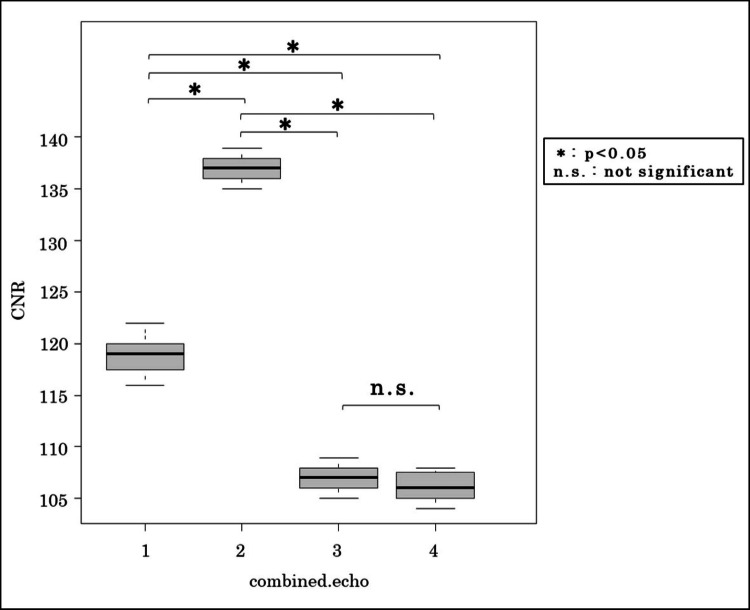
IGHL-subscapularis muscle CNR at a 5° flip angle The highest CNR was observed with a two-echo combination. Statistical significance was determined using the Wilcoxon rank-sum test with Bonferroni correction. CNR, contrast-to-noise ratio; IGHL, inferior glenohumeral ligament

Figure 4 displays the CNRs between the IGHL and subscapularis muscle at a 5° flip angle, where no signal inversion occurred. The highest CNR (137.1) was achieved with two combined echoes, with the following CNR values for echoes 1-4: 118.8, 137.1, 106.8, 106.1. Statistical comparisons using the Wilcoxon rank-sum test with Bonferroni correction revealed a significant difference between the 2-echo condition and the other groups (p < 0.05).

Optimal readout gradient polarity

**Figure 5:**
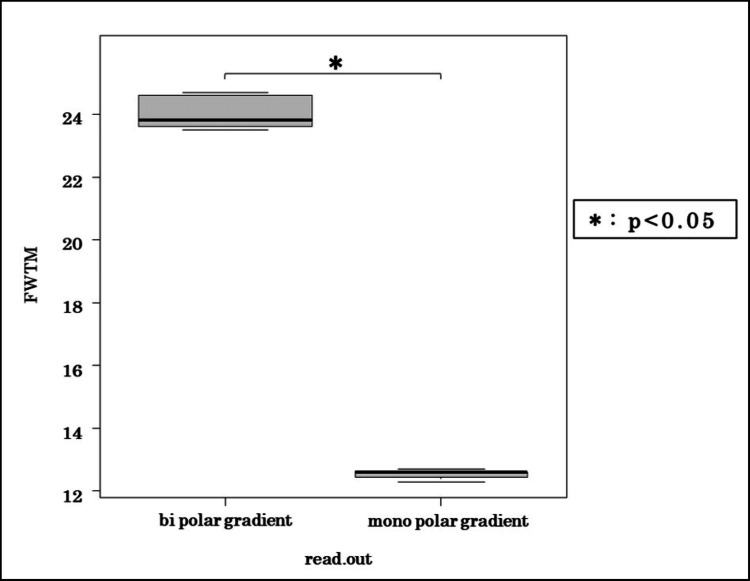
FWTM analysis comparing monopolar and bipolar gradients The monopolar gradient produced significantly narrower signal profiles, indicating less blurring. FWTM, full width at tenth maximum

Figure 5 compares IGHL profile curves obtained under monopolar and bipolar readout gradients. The bipolar gradient produced broader and less steep curves, with FWTM values 47% higher than those obtained with the monopolar gradient. Wilcoxon testing confirmed that this difference was statistically significant (p < 0.05).

Usefulness of subtraction imaging

**Figure 6:**
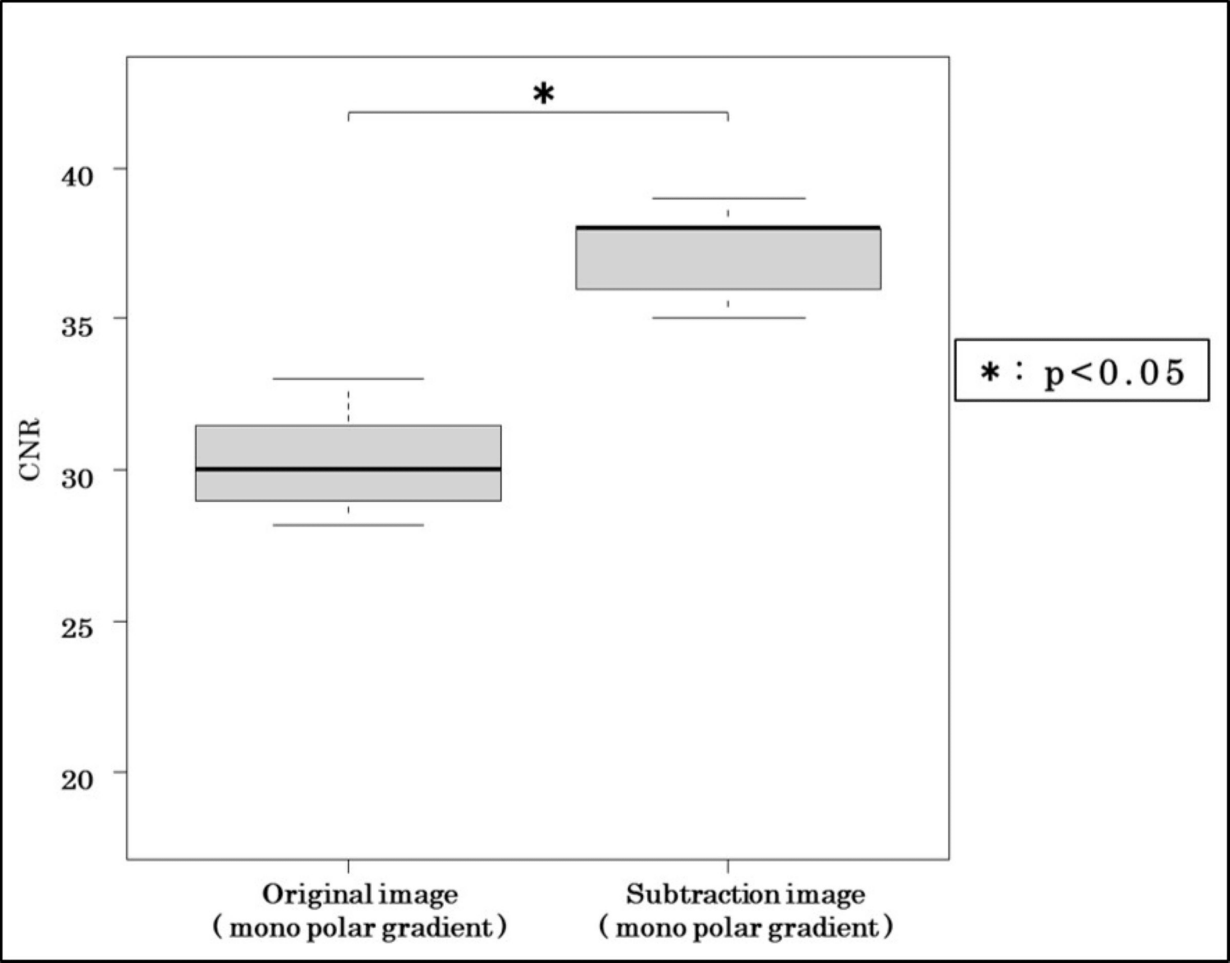
CNR comparison between original and subtraction images Subtraction processing improved the CNR by 15.8%. CNR, contrast-to-noise ratio

**Figure 7:**
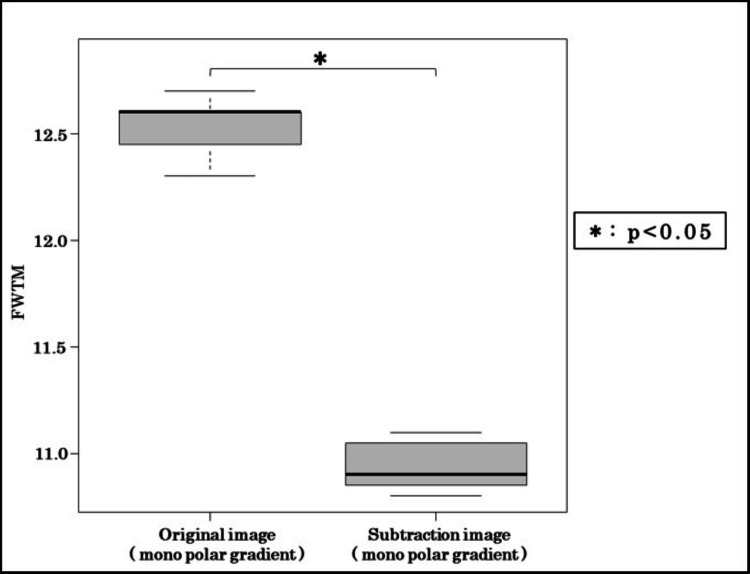
FWTM comparison between original and subtraction images Subtraction processing reduced FWTM by 11.2%, indicating enhanced sharpness. FWTM, full width at tenth maximum

Figure 6 and Figure 7 compare monopolar original and subtraction images. Subtraction processing increased the IGHL-subscapularis CNR by 15.8% (original: 137.1 vs. subtraction: 158.7) and reduced FWTM by 11.2%, suggesting sharper ligament edges. Wilcoxon testing revealed that both metrics were significantly improved by subtraction (p < 0.05).

Visual assessment

**Figure 8:**
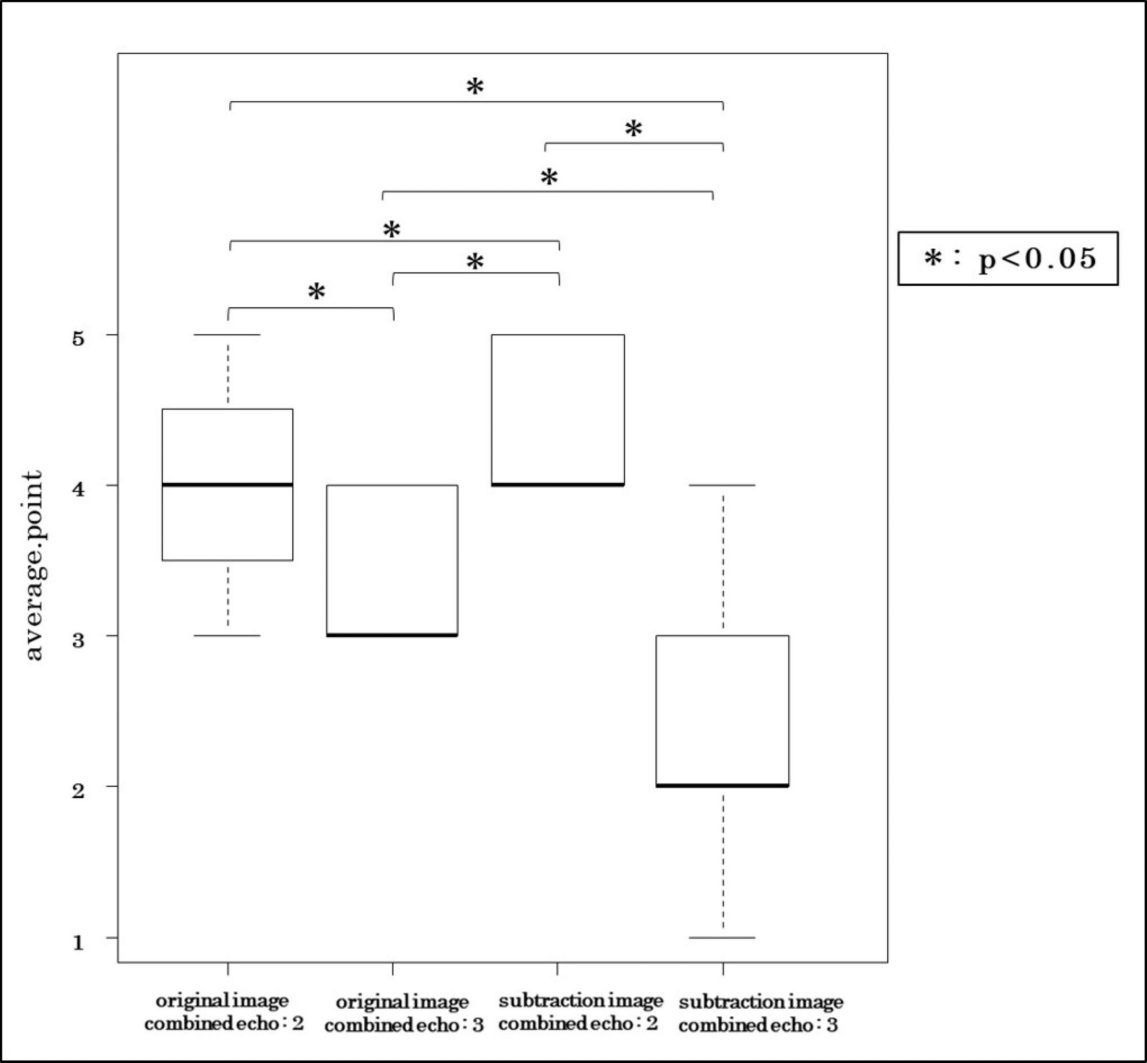
Visual assessment scores across different imaging conditions Box-and-whisker plots display the median, IQR, minimum/maximum, and outliers.

Figure 8 presents visual assessment scores across different imaging conditions. The 5°/2-echo images with subtraction processing received the highest scores (mean ± SD: 4.73 ± 0.45), while subtraction images from three-echo combinations were rated significantly lower (2.87 ± 0.83). Wilcoxon rank-sum testing with Bonferroni correction confirmed statistical significance (p < 0.01).

Clinical case: bone and ligament visualization

**Figure 9:**
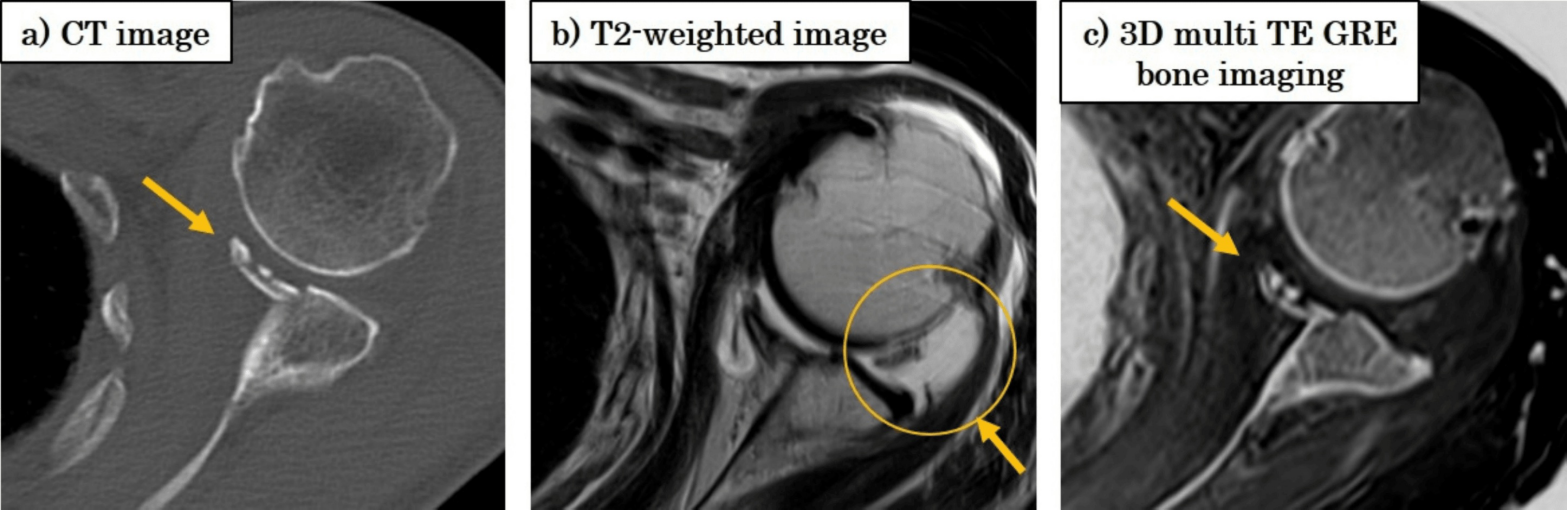
Clinical case: anteroinferior glenoid bone defect (a) CT shows the bone defect; (b) MRI shows joint effusion; (c) CT-like MRI provides a clearer depiction of the defect.

**Figure 10:**
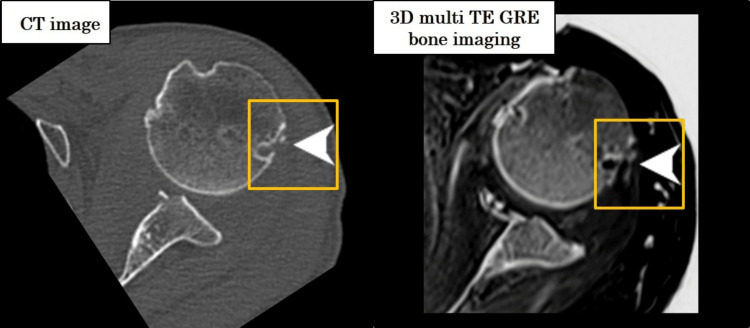
Clinical case: Hill-Sachs lesion CT-like MRI clearly visualizes the humeral head impaction fracture.

**Figure 11:**
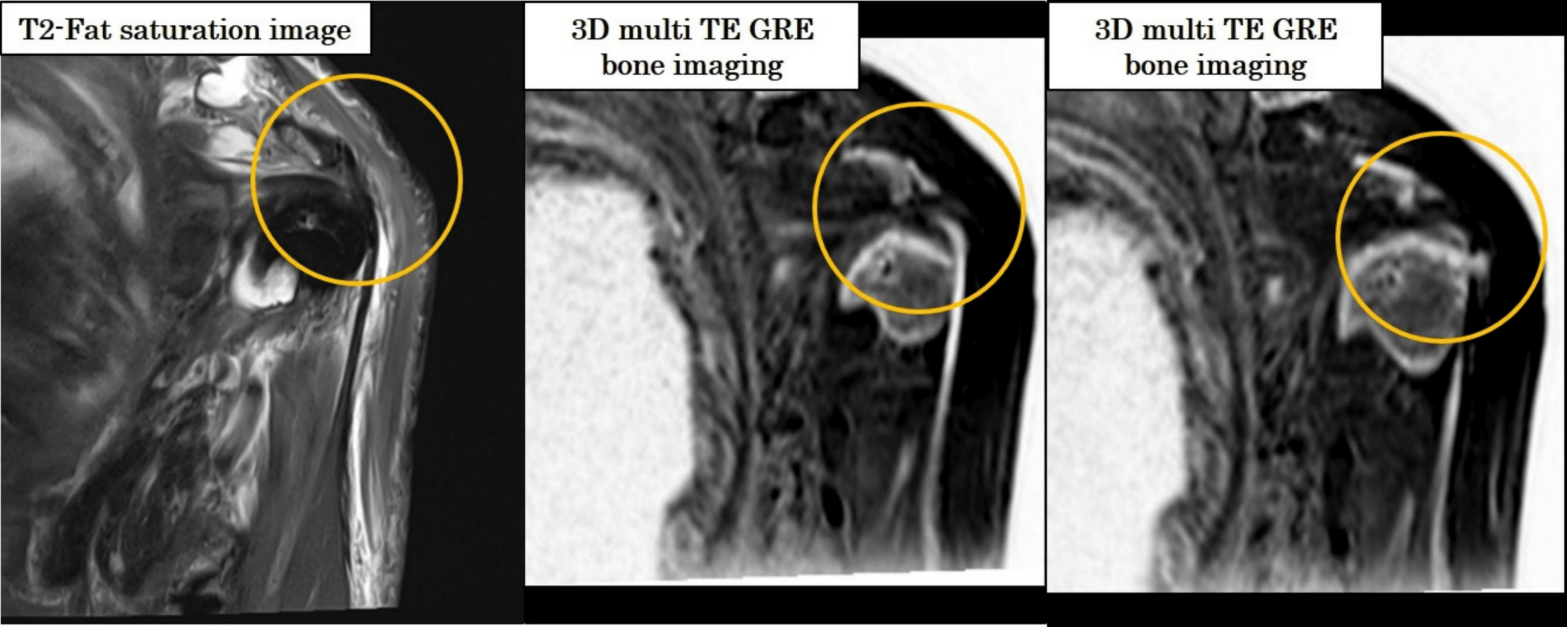
Long head of the biceps tendon attachment CT-like MRI delineates the attachment site despite fluid signal interference.

Figure 9, Figure 10, and Figure 11 demonstrate clinical application in a case of anterior shoulder dislocation. CT (Figure 9a) revealed an anteroinferior glenoid bone defect, while MRI (Figure 9b) showed a fluid signal obscuring the defect. CT-like MRI (Figure 9c) suppressed the fluid signal and clearly delineated the bone defect.

A Hill-Sachs lesion was clearly visualized in the CT-like MRI (Figure 10), and the attachment of the long head of the biceps tendon was well depicted (Figure 11), consistent with fat-suppressed T2-weighted MRI. Furthermore, the Hill-Sachs lesion was distinctly visible, similar to the findings on CT (Figure 10, arrow).

On the T2-weighted fat-suppressed images, high signal intensity was observed at the attachment site of the long head of the biceps tendon, although no tear was identified. CT-like MRI successfully depicted this attachment site as well (Figure 11, circle).

## Discussion

CT-like imaging using 3D GRE sequences with multi-echo acquisition allows for the indirect visualization of tissues with short T2* values through a grayscale inversion process. This technique builds on prior work, including the FRACTURE method introduced by Johnson et al. [12], which uses fast field echo imaging with restricted echo spacing to achieve CT-like contrast via grayscale inversion. The method capitalizes on GRE-specific characteristics, where T1-weighted contrast increases with larger flip angles [14]. Fat-containing tissues, such as cancellous bone, are particularly sensitive to T1 weighting, exhibiting higher signal intensity at larger flip angles [15]. Consequently, at flip angles ≥10°, signal inversion was observed, with trabecular bone appearing brighter than the subscapularis muscle, leading to reduced contrast and diminished diagnostic accuracy. Therefore, it is crucial to use lower flip angles to avoid signal inversion in target structures.

**Figure 12:**
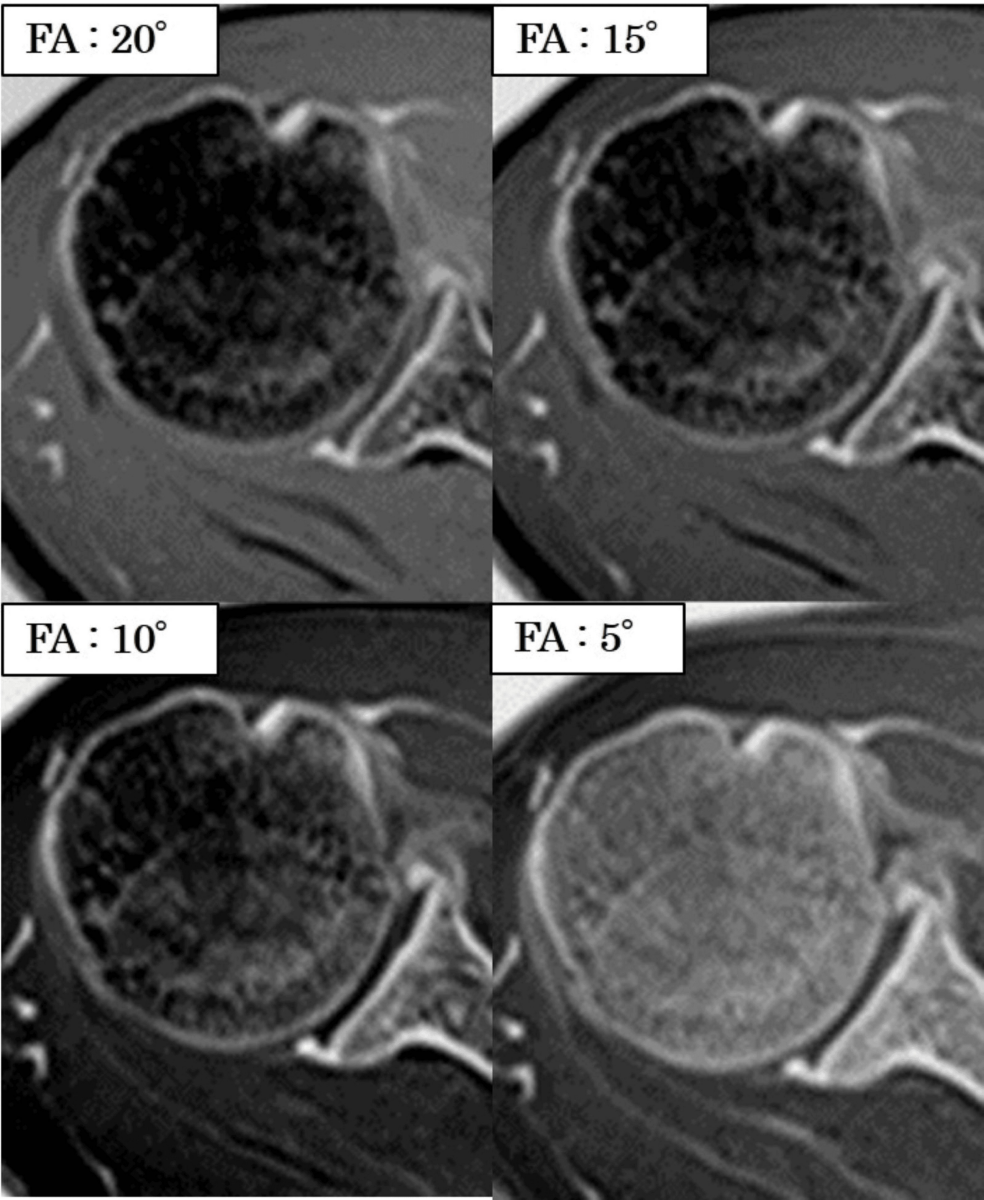
Signal inversion due to flip angle variations in grayscale-inverted CT-like MRI Representative images show the effect of flip angle on signal intensity in grayscale-inverted images. At a 5° flip angle, trabecular bone appears hypointense relative to muscle in the original image and appropriately hyperintense after inversion. At flip angles ≥10°, trabecular bone signal exceeds that of muscle before inversion, resulting in reduced contrast and potential misinterpretation in the inverted image. This highlights the importance of using lower flip angles to avoid signal inversion in fat-containing tissues.

Figure 12 presents a visual comparison of signal inversion at different flip angles.

A critical aspect of grayscale inversion is that tissues appearing hypointense in the original image become hyperintense in the inverted image. For effective visualization of bone and ligament structures, these tissues must exhibit relatively low signal intensity compared to surrounding tissues before inversion. Our results demonstrated that using a 5° flip angle and two combined echoes consistently produced low signal intensity in cortical bone, trabecular bone, and the IGHL, enhancing contrast and delineation in the inverted image. These findings are consistent with signal intensity measurements, CNR values, and visual assessment scores.

Echo combination analysis revealed that increasing the number of combined echoes progressively suppressed water signals due to the inclusion of longer TE components [16]. This resulted in a general decrease in signal intensity across both trabecular bone and muscle, reducing contrast between these structures. In contrast, the two-echo combination preserved tissue contrast and provided clearer anatomical delineation.

**Figure 13:**
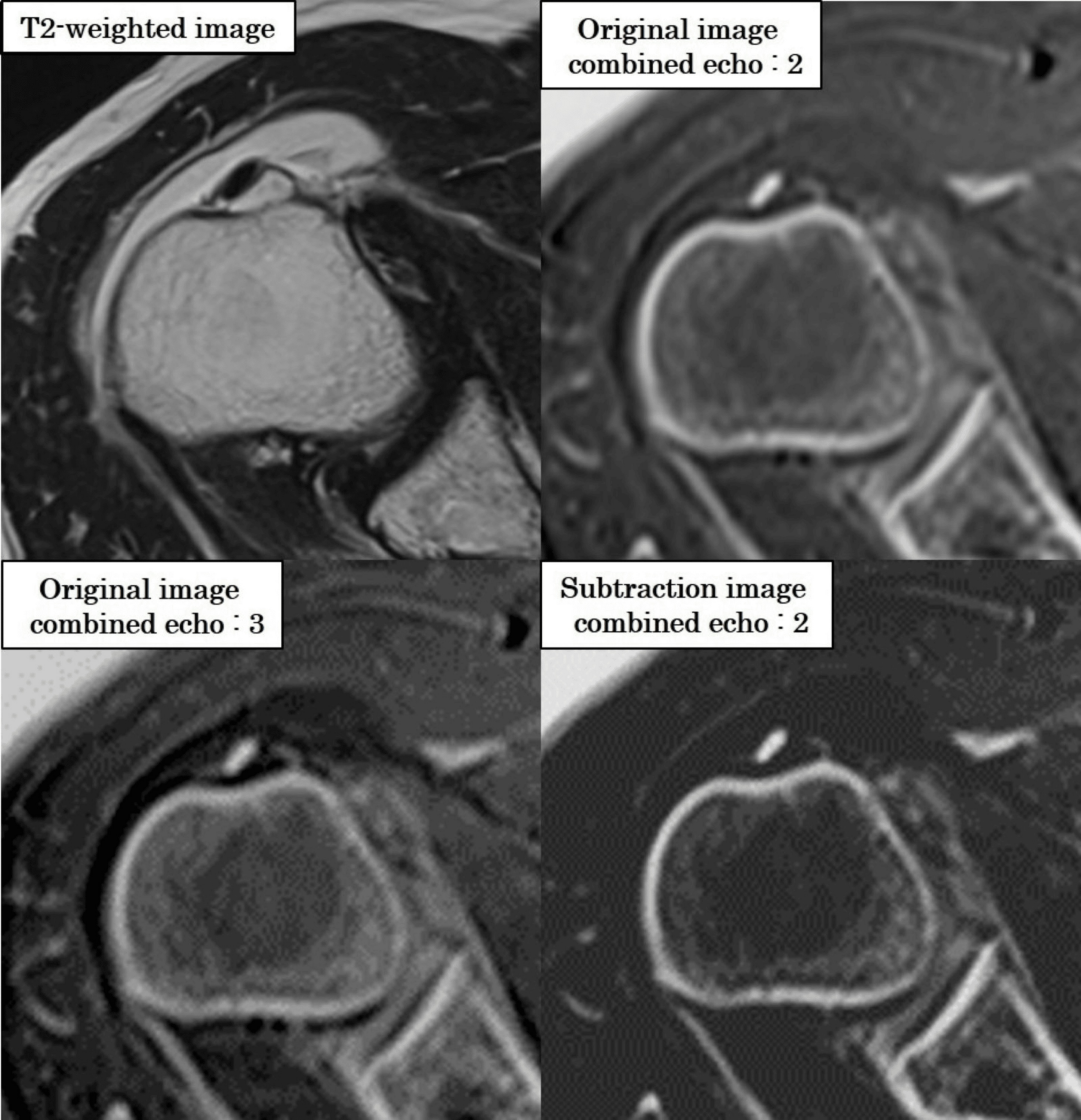
Suppression of water signal with increased number of combined echoes in grayscale-inverted CT-like MRI Representative images show how increasing the number of combined echoes affects signal characteristics. As the number of combined echoes increases (from one to three), the water signal becomes more suppressed due to the inclusion of longer TE components. While this enhances background suppression, it may also reduce contrast between bone and muscle, potentially compromising structural delineation in grayscale-inverted images.

Figure 13 illustrates the effect of increasing the number of combined echoes on water signal suppression.

Subtraction processing further enhanced image quality by suppressing background signals while preserving tissue-specific contrast. The results of CNR and FWTM analyses, along with visual scoring, confirmed that the 5°/2-echo subtraction condition yielded the highest image quality. These findings also clarify that averaging long-TE components, while effective at water suppression, may inadvertently reduce grayscale-inverted contrast due to enhanced fluid signals.

Visual assessment confirmed these technical results, with the 5°/2-echo subtraction images receiving the highest scores. In contrast, three-echo subtraction images exhibited reduced contrast and structural clarity, underscoring the need to balance echo combination and contrast enhancement for optimal CT-like MRI performance.

**Figure 14:**
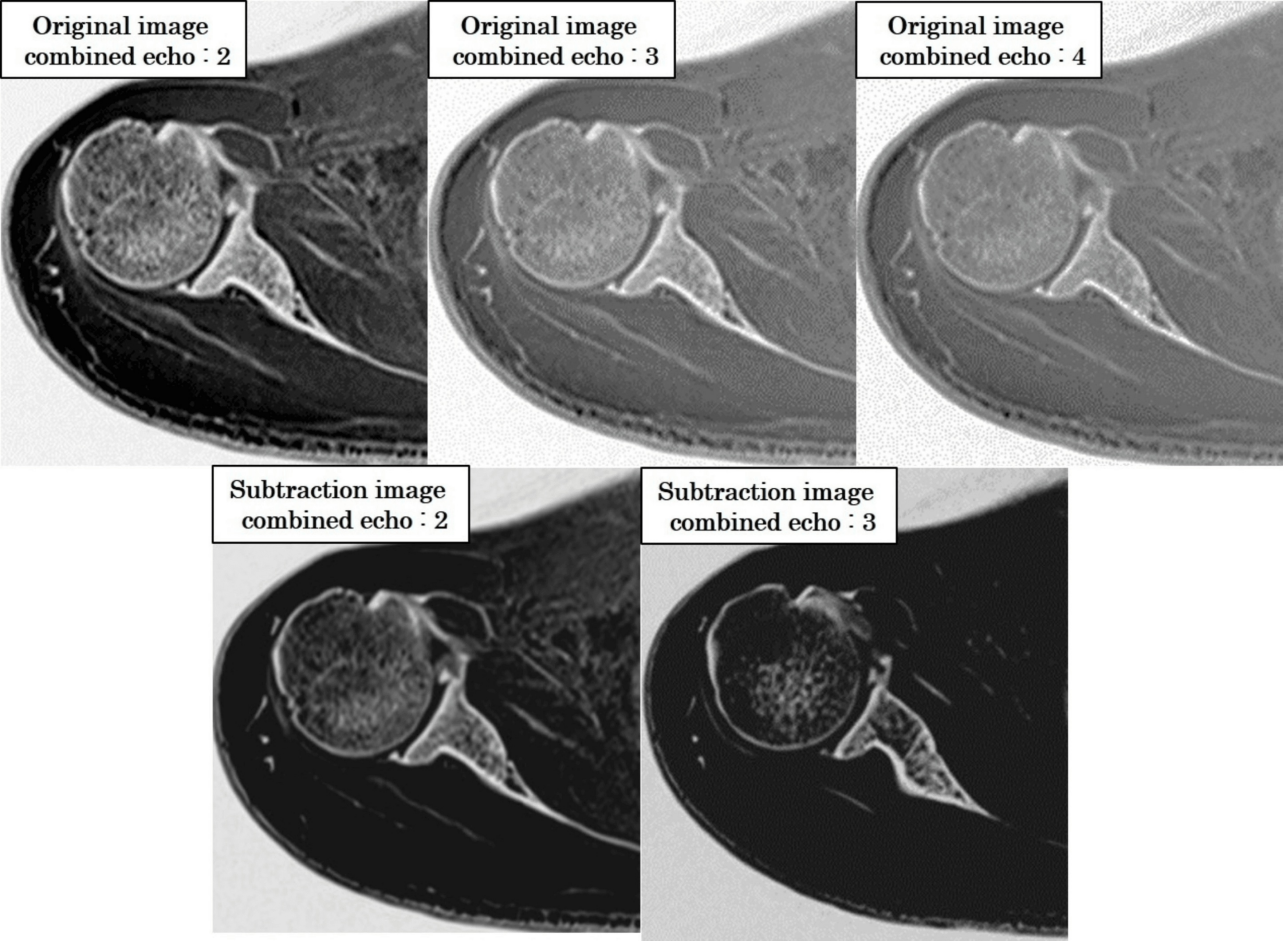
Effect of echo combination and subtraction processing on image contrast in CT-like MRI Grayscale-inverted images generated using two, three, and four combined echoes, as well as subtraction images based on two- and three-echo combinations. The two-echo subtraction condition yielded the highest bone-to-muscle contrast and the most distinct anatomical delineation. In comparison, three- and four-echo images showed progressively lower contrast due to signal averaging, and three-echo subtraction images also exhibited diminished tissue differentiation. Visual assessment confirmed the superiority of the two-echo subtraction approach for CT-like image quality.

Figure 14 presents subtraction processing outcomes across different echo combinations.

In a clinical case of anterior shoulder dislocation, CT-like MRI successfully depicted glenoid bone loss and a Hill-Sachs lesion while effectively suppressing joint effusion signals. This supports its clinical utility, especially in situations where conventional MRI may obscure osseous or ligamentous details due to fluid interference.

While our findings are promising, they are limited by the inclusion of only one clinical case, which restricts generalizability. Furthermore, the study was conducted at a single center using a single 1.5T MRI system, which may limit the external validity of the results. To minimize unnecessary CT exposure, participants were limited to those aged ≤50 years, introducing potential age-related bias. This age restriction was implemented to ethically justify omitting CT in healthy volunteers, who are more sensitive to cumulative radiation exposure. Additionally, the use of only one clinical case limits our ability to draw broad diagnostic conclusions. Future studies should involve multi-institutional validation, the use of equipment from different vendors, a broader age range, and a more diverse set of clinical cases to fully establish the clinical potential of CT-like MRI.

## Conclusions

In our study, CT-like imaging using a 3D multi-echo GRE sequence with a 5° flip angle and two combined echoes successfully visualized bone and ligament structures in the shoulder. Image blurring was reduced through the use of a monopolar readout gradient, and contrast was enhanced by subtraction processing. This technique does not involve radiation exposure and was applied to assess bone and ligament anatomy in a clinical case of anterior shoulder dislocation.
